# Supplementary material for: Content-rich biological network constructed by mining PubMed abstracts
Source: BMC Bioinformatics. 2004 Oct 8;5:147. doi: 10.1186/1471-2105-5-147 (PMC528731; doi:10.1186/1471-2105-5-147)
Supplement: Additional File 2 — The original results of the above study (non-essential files are deleted to keep the file size under the limit set by BMC bioinformatics). [file 1471-2105-5-147-S2.bz2 › chilibotAdditionalFile2/dip20/213ID7790364E719/html/ITGA3_CD9.html]

 


 **ITGA3** and **CD9** 
  
Found 15 abstracts in PubMed, retrieved 15.  
 

 What does Google say? 
 PDF only 
| .edu only 

---

**Interactive relationship** (e.g. stimulation, inhibition, etc)

**Neutral relationship**- Integrin alpha3  [ **ITGA3** ]  gene expression was independent from MRP 1  [ **CD9** ]   **CD9**  gene expression.  Ref: 9508191 J Clin Oncol, 1998
- Anti integrin alpha 3  [ **ITGA3** ]  or anti integrin beta 1 coprecipitated DRAP27  [ **CD9** ]   **CD9**  from the same cell lysates.  Ref: 7790364 J Cell Biol, 1995
- Membrane anchored heparin binding EGF like growth factor HB EGF and diphtheria toxin receptor associated protein DRAP27  [ **CD9** ]   **CD9**  form a complex with integrin alpha 3  [ **ITGA3** ]  beta 1 at cell cell contact sites.  Ref: 7790364 J Cell Biol, 1995
- **CD9**  is expressed in extravillous trophoblasts in association with integrin alpha3  [ **ITGA3** ]  and integrin alpha5.  Ref: 10065872 Mol Hum Reprod, 1999
- Immunoprecipitation with anti DRAP27  [ **CD9** ]   **CD9**  resulted in preferential coprecipitation of integrin alpha 3  [ **ITGA3** ]  beta 1 from Vero cell, A431 cell and MG63 cell lysates.  Ref: 7790364 J Cell Biol, 1995

**Non-interactive relationship** (e.g. studied together, co-existance, homology, etc.)

- Chemical cross linking confirmed the physical association of DRAP27  [ **CD9** ]   **CD9**  and integrin alpha 3  [ **ITGA3** ]  beta 1.  Ref: 7790364 J Cell Biol, 1995
- The loss of MRP1  [ **CD9** ]   **CD9**  and integrin alpha3  [ **ITGA3** ]  has been involved in tumor growth and metastasis of cancer cells.  Ref: 11505398 Cancer, 2001
- PATIENTS AND METHODS We performed a retrospective study of integrin alpha3  [ **ITGA3** ]  and MRP 1  [ **CD9** ]   **CD9**  expression in resected tumor tissues from 151 NSCLC patients using quantitative reverse transcriptase polymerase chain reaction RT PCR and immunohistochemistry.  Ref: 9508191 J Clin Oncol, 1998
- The aim of the current study was to clarify the clinical significance of MRP1  [ **CD9** ]   **CD9**  and integrin alpha3  [ **ITGA3** ]  in endometrial cancer.  Ref: 11505398 Cancer, 2001
- Data obtained are presented in concurrence with Clusters of Thymic Epithelial Staining CTES classification for various monoclonal antibodies recognizing CD antigens CD1, CD1a, CD6,  **CD9** , CD14, CD16, CD29, CD30, CD32, CD44, CD45RB, CD47, CD48, CD49a, CD49b, CD49c  [ **ITGA3** ] , CD49d, CD49e, CD49f, CD51, CD53, CD54, CD56, CD57, CD63, CD85, CD95, CD98, CD102, CD103, CD106, CD109, CD146, CD147, CD148, CD151, CD152, CD158a, CD158b, CD164, CD165, CD166 and for monoclonal antibodies 1B1 G7, A4, BD46, BLTZ, HP1C5, IND.64, M72, WU947 whose specifities are not yet defined.  Ref: 12725440 Ann Anat, 2003
- PURPOSE We investigated the possible association between integrin alpha3  [ **ITGA3** ]  and motility related protein  [ **CD9** ]  MRP 1  [ **CD9** ] , cluster of differentiation antigen 9  **CD9**  gene expression in non small cell lung cancer NSCLC and evaluated the prognostic significance of integrin alpha3  [ **ITGA3** ]  expression.  Ref: 9508191 J Clin Oncol, 1998
- Integrin alpha3  [ **ITGA3** ]  expression as a prognostic factor in colon cancer association with MRP 1  [ **CD9** ]   **CD9**  and KAI1 CD82.  Ref: 11802216 Int J Cancer, 2002
- RESULTS HB EGF, its receptors HER 1 and HER 4, and the associated proteins  **CD9** , CD44, and integrin alpha 3  [ **ITGA3** ]  beta 1 were expressed by HPMCs and peritoneal macrophages.  Ref: 11168942 Kidney Int, 2001
- Loss of motility related protein  [ **CD9** ]  1 MRP1  [ **CD9** ]   **CD9**  and integrin alpha3  [ **ITGA3** ]  expression in endometrial cancers.  Ref: 11505398 Cancer, 2001
- RESULTS In normal endometrium, MRP  **CD9**  was expressed at the cell membrane of cell contact sites, and the expression of integrin alpha3  [ **ITGA3** ]  was detected also at the cell membrane of cell contact sites and at borders of stromal tissues.  Ref: 11505398 Cancer, 2001
- At cell cell contact sites, DRAP27  [ **CD9** ]   **CD9**  was highly coincident with alpha catenin and vinculin, suggesting that DRAP27  [ **CD9** ]   **CD9** , proHB EGF, and integrin alpha 3  [ **ITGA3** ]  beta 1 are colocalized with adherence junction locating proteins.  Ref: 7790364 J Cell Biol, 1995
- Disease free survival curves were estimated using the Kaplan Meier method and analyzed by the log rank test between the positive and reduced expression statuses of both MRP1  [ **CD9** ]   **CD9**  and integrin alpha3  [ **ITGA3** ] .  Ref: 11505398 Cancer, 2001
- Immunoprecipitation followed by Western blot analysis showed association between  **CD9**  and integrin alpha3  [ **ITGA3** ]  in endometrium.  Ref: 12021064 Biol Reprod, 2002
- CONCLUSIONS These findings suggested that the analysis for the expression statuses of MRP1  [ **CD9** ]   **CD9**  and integrin alpha3  [ **ITGA3** ]  may provide important information on the clinical behavior of endometrial cancer.  Ref: 11505398 Cancer, 2001
- Anti  **CD9** , CD26, CD29, CD31, CD36, CD44, CD49a, CD49b, CD49c  [ **ITGA3** ] , CD49d, CD49e, CD49f, CD54, CD55, CD56, CD62E &.  Ref: 10235320 J Pediatr Surg, 1999
- METHODS The expression of MRP1  [ **CD9** ]   **CD9**  and integrin alpha3  [ **ITGA3** ]  from the same tissue sample were examined immunohistochemically in 15 patients with normal endometrium and in 56 patients with uterine endometrioid adenocarcinoma.  Ref: 11505398 Cancer, 2001
- Fourteen cases indicated a reduced expression of both MRP1  [ **CD9** ]   **CD9**  and integrin alpha3  [ **ITGA3** ] .  Ref: 11505398 Cancer, 2001
- A correlation was found between integrin alpha3  [ **ITGA3** ]  with MRP 1  [ **CD9** ]   **CD9**  and KAI1 CD82 for stage I tumors.  Ref: 11802216 Int J Cancer, 2002
- colon cancer progresses following collapse of the complex formed by integrin alpha3  [ **ITGA3** ]  with MRP 1  [ **CD9** ]   **CD9**  and KAI1 CD82.  Ref: 11802216 Int J Cancer, 2002
- CONCLUSION No significant association was found between integrin alpha3  [ **ITGA3** ]  and MRP 1  [ **CD9** ]   **CD9**  gene expression in lung cancer.  Ref: 9508191 J Clin Oncol, 1998
- In patients with endometrioid adenocarcinoma, 17 cases showed reduced expression of MRP1  [ **CD9** ]   **CD9** , and 20 cases had reduced expression of integrin alpha3  [ **ITGA3** ] .  Ref: 11505398 Cancer, 2001
- Each reduced expression of MRP1  [ **CD9** ]   **CD9**  or integrin alpha3  [ **ITGA3** ]  was significantly correlated with histologic grade and metastasis.  Ref: 11505398 Cancer, 2001
- Using Vero H cells, which overexpress HB EGF, we also demonstrated the association of proHB EGF with DRAP27  [ **CD9** ]   **CD9**  and integrin alpha 3  [ **ITGA3** ]  beta 1.  Ref: 7790364 J Cell Biol, 1995
- BACKGROUND MRP1  [ **CD9** ]   **CD9**  and integrin alpha3  [ **ITGA3** ]  have played crucial roles in cell adhesion, motility, and signaling events.  Ref: 11505398 Cancer, 2001
- Furthermore, we investigated the correlation of integrin alpha3  [ **ITGA3** ]  with MRP 1  [ **CD9** ]   **CD9**  and KAI1 CD82.  Ref: 11802216 Int J Cancer, 2002
- Moreover, colocalization of proHB EGF, DRAP27  [ **CD9** ]   **CD9** , and integrin alpha 3  [ **ITGA3** ]  beta 1 at cell cell contact sites was observed by double immunofluorescence staining.  Ref: 7790364 J Cell Biol, 1995
- Proteins were purified from chorion laeve by affinity chromatography with anti integrin alpha3  [ **ITGA3** ]  and alpha5 monoclonal antibodies and Western blotting, revealed that  **CD9**  was associated with both integrins.  Ref: 10065872 Mol Hum Reprod, 1999
